# Rare variants analysis of cutaneous malignant melanoma genes in Parkinson's disease

**DOI:** 10.1016/j.neurobiolaging.2016.07.013

**Published:** 2016-12

**Authors:** S.J. Lubbe, V. Escott-Price, A. Brice, T. Gasser, A.M. Pittman, J. Bras, J. Hardy, P. Heutink, N.M. Wood, A.B. Singleton, D.G. Grosset, C.B. Carroll, M.H. Law, F. Demenais, M.M. Iles, Melanoma Meta-Analysis Consortium, D.T. Bishop, J. Newton-Bishop, N.M. Williams, H.R. Morris

**Affiliations:** a Department of Clinical Neuroscience, UCL Institute of Neurology, London, United Kingdom; b Institute of Psychological Medicine and Clinical Neurosciences, Medical Research Council Centre for Neuropsychiatric Genetics and Genomics, Department of Psychological Medicine and Neurology, Cardiff University School of Medicine, Cardiff, United Kingdom; c INSERM U 1127, CNRS UMR 7225, Sorbonne Universités, UPMC Univ Paris 06 UMR S 1127, Institut du Cerveau et de la Moelle Epinière, ICM, France; d Department for Neurodegenerative Diseases, Hertie Institute for Clinical Brain Research, and DZNE, German Center for Neurodegenerative Diseases, Tübingen, Germany; e Department of Molecular Neuroscience, UCL Institute of Neurology, London, United Kingdom; f Department of Clinical Genetics, Section of Medical Genomics, VU University Medical Centre, Amsterdam, The Netherlands; g UCL Genetics Institute, London, United Kingdom; h Laboratory of Neurogenetics, National Institute on Aging, National Institutes of Health, Bethesda, MD, USA; i Department of Neurology, Institute of Neurological Sciences, Queen Elizabeth University Hospital, Glasgow, United Kingdom; j Plymouth University Peninsula Schools of Medicine and Dentistry, Plymouth, United Kingdom; k Statistical Genetics, QIMR Berghofer Medical Research Institute, Brisbane, Queensland, Australia; l INSERM, UMR 946, Genetic Variation and Human Diseases Unit, Paris, France; m Institut Universitaire d'Hématologie, Université Paris Diderot, Sorbonne, Paris, France; n Section of Epidemiology and Biostatistics, Leeds Institute of Cancer and Pathology, Leeds, United Kingdom

**Keywords:** Parkinson's, Cutaneous malignant melanoma, Shared genetic background, Pigmentation, Tyrosinase

## Abstract

A shared genetic susceptibility between cutaneous malignant melanoma (CMM) and Parkinson's disease (PD) has been suggested. We investigated this by assessing the contribution of rare variants in genes involved in CMM to PD risk. We studied rare variation across 29 CMM risk genes using high-quality genotype data in 6875 PD cases and 6065 controls and sought to replicate findings using whole-exome sequencing data from a second independent cohort totaling 1255 PD cases and 473 controls. No statistically significant enrichment of rare variants across all genes, per gene, or for any individual variant was detected in either cohort. There were nonsignificant trends toward different carrier frequencies between PD cases and controls, under different inheritance models, in the following CMM risk genes: *BAP1*, *DCC*, *ERBB4, KIT*, *MAPK2*, *MITF*, *PTEN*, and *TP53*. The very rare *TYR* p.V275F variant, which is a pathogenic allele for recessive albinism, was more common in PD cases than controls in 3 independent cohorts. Tyrosinase, encoded by *TYR,* is the rate-limiting enzyme for the production of neuromelanin, and has a role in the production of dopamine. These results suggest a possible role for another gene in the dopamine-biosynthetic pathway in susceptibility to neurodegenerative Parkinsonism, but further studies in larger PD cohorts are needed to accurately determine the role of these genes/variants in disease pathogenesis.

## 1 Introduction

Parkinson's disease (PD) is characterized by the progressive loss of postmitotic dopaminergic neurons, whereas cancer results from uncontrolled cellular proliferation. Although PD and cancer are distinct diseases, a relationship between PD and cancer is well established. Epidemiological studies have shown that although most cancers are less frequent in PD compared with the general population (Bajaj et al., 2010, Becker et al., 2010, Catalá-López et al., 2014, D'Amelio et al., 2004, Elbaz et al., 2002, Elbaz et al., 2005; Gao et al., 2009a; Kareus et al., 2012, Olsen et al., 2005, Olsen et al., 2006, Ong et al., 2014, Wirdefeldt et al., 2014), cutaneous malignant melanoma (CMM) is found at an increased incidence in PD (Bajaj et al., 2010, Becker et al., 2010, Catalá-López et al., 2014, Kareus et al., 2012, Ong et al., 2014, Wirdefeldt et al., 2014). This well-documented association between CMM and PD is unexplained.

A genetic link between PD and CMM is supported by the demonstration of significant reciprocal risks of PD and CMM in cases and their relatives (Gao et al., 2009a, Gao et al., 2009b, Kareus et al., 2012). Although some support for a somatic genetic link between the 2 pathologies is provided by the role of Mendelian PD genes in CMM biology (Cesari et al., 2003, Kim et al., 2005, Liu et al., 2011, Matsuo and Kamitani, 2010, Millikin et al., 1991), there is currently no direct evidence for shared genetic susceptibility between PD and CMM.

Some studies have assessed the reciprocal role of common (minor allele frequency [MAF] > 1%) genetic variation in CMM and PD. Recently, it has been suggested that the CMM-associated *MC1R* variants p.R151C and p.R160W increase PD risk but their role still remains unclear (Dong et al., 2014, Gao et al., 2009b, Lubbe et al., 2016, Tell-Marti et al., 2015). Previous studies using genome-wide association study variants associated with PD or CMM have failed to show any genetic overlap (Dong et al., 2014, Meng et al., 2012). More recently, rare de novo variants in the CMM risk gene *PTEN* have been implicated in PD (Kun-Rodrigues et al., 2015), but the role of rare coding variants underlying an association between PD and CMM has not yet been fully evaluated. Because the role of common genetic variation (variants with MAF >1%) has already been substantially addressed, we focused our investigation into the proposed shared genetic background between these diseases on rare variants (MAF <1%) in known CMM genes in 2 large independent PD case-control data sets as part of the International Parkinson's Disease Genomics Consortium.

## 2 Methods and materials

### 2.1 Genetic analysis

Using a systematic literature search, we identified susceptibility genes for CMM (Supplementary Table 1). These included (1) germline high-risk genes associated with familial CMM (e.g., *CDKN2A*, *CDK4*); (2) germline common moderate-risk genes (e.g., *MC1R*); (3) genes commonly somatically mutated (e.g., *BRAF*); and (4) recently identified genes found to harbor rare somatic mutations ascribed to CMM (e.g., *TRRAP*, *DCC*). Genes were selected based on defined roles in inherited high-penetrance autosomal dominant disease (n = 2); an excess of somatic mutations (n = 20); an excess of common low-penetrance risk variants (n = 3); or combinations of these (n = 4). All rare (MAF <1%) variants across these genes were assessed for enrichment in PD cases compared with unaffected controls.

We first assessed high-quality rare variant genotype data derived from the NeuroX chip on 6875 PD cases and 6065 controls (dbGaP Study Accession: phs000918.v1.p1). Briefly, the NeuroX chip has approximately 240,000 preselected variants based on standard Illumina exome content and over 24,000 custom content neurologic disease focused variants (Nalls et al., 2015).

We next assessed whole-exome sequencing data on 1255 PD cases and 473 controls from the International Parkinson's Disease Genomics Consortium. Briefly, sample libraries from cases and controls were prepared using either Roche Nimblegen (cases, n = 334; controls, n = 40) or Illumina (cases, n = 921; controls, n = 433) capture kits with paired-end sequencing performed on the Illumina HiSeq2000. Reads were aligned using Burrows-Wheeler Aligner (Li and Durbin, 2009) against the University of California Santa Cruz (UCSC) hg19 reference genome. Variant calling and quality-based filtering were done using Genome Analysis Tool Kit (GATK) (McKenna et al., 2010). ANNOVAR (Wang et al., 2010) was used to annotate variants with predicted impact of variants from the following in silico tools: SIFT (Ng and Henikoff, 2001), PhyloP (Pollard et al., 2010), PolyPhen-2 (Adzhubei et al., 2010), LRT (Chun and Fay, 2009), MutationTaster (Schwarz et al., 2010), and GERP++ (Davydov et al., 2010).

Of the 29 identified CMM genes, only 24 were represented on the NeuroX panel (Supplementary Table 1). Based on the annotated MAF data from 1000 Genomes Project (http://www.1000genomes.org/) and NHLBI GO Exome Sequencing Project (https://evs.gs.washington.edu/EVS/), all rare variants (MAF < 1%) were extracted and assessed in PD cases and controls. We defined the potential deleterious impact of variants using previously defined methods (Fu et al., 2013, Tennessen et al., 2012) with variants classified as damaging if ≥4 of the 6 in silico tools used predicted the change deleterious. Variants and samples with >5% missing calls were excluded during QC.

All exome generated FastQs were run through the same pipeline and merged to generate high-quality genotype data. Damaging variants were defined as stated above. The GATK recommended filtering of variants, including the removal of variants with low coverage (read depth <5), was implemented over and above the QC stated above. Post QC, 28 of the 29 selected CMM genes were covered by one or both captures methods (Supplementary Table 1), and no difference between capture methods was observed with majority of all exons represented and included in the analyses (Supplementary Table 2).

Candidate variants were also assessed in high-quality exome sequencing data generated from a CMM case-control cohort (CMM, n = 1298; Controls, n = 684) to investigate any reciprocal risks for CMM.

### 2.2 Statistical analysis

SNP-Set (Sequence) Kernel Association Test (SKAT) (Wu et al., 2011) was used to test for association between the rare variants in genes and PD (gene- and gene set-based), adjusting for covariates including gender, coverage metrics and principal components (1–4). Dominant and recessive models of inheritance for each CMM gene were modeled and assessed using STATA (version 10; STATA, State College, TX, USA) via logistic regression, adjusting for covariates. For variants common to both cohorts, meta-analyses were conducted using standard methods modeling fixed effects (Petitti, 1994). Cochran's Q-statistic was calculated to test for heterogeneity (P_het_) (Petitti, 1994), and the I^2^ statistic (Higgins and Thompson, 2002) was generated to quantify the proportion of the total variation caused by heterogeneity. Bonferroni's correction was applied, where applicable, to account for multiple testing.

## 3 Results

### 3.1 Rare variant screening and burden analysis

The NeuroX data contained 237 variants with ≥1 nonreference allele after QC, including 215 (90.7%) nonsynonymous single nucleotide polymorphisms (nsSNPs), and 17 (7.2%) loss of function (LOF) variants (stop gains or losses, splice, frame- or nonframeshift indels). About 554 variants with ≥1 nonreference allele were extracted from exome sequencing data, including 268 (48.4%) synonymous, 269 (48.6%) nsSNPs, and 17 (3.0%) LOF variants. In the NeuroX data, 207 rare (MAF <1%) variants were present in 23 of the 29 selected CMM genes, and 269 rare variants were observed in 25 genes in the exome.

When considering all the candidate genes at once, gene set–based SKAT analyses in the NeuroX data did not identify any significant difference in rare variation burden between PD cases and controls for all variants, or for each individual variant class (Table 1).

When considering all variation (including synonymous) within each gene individually (Supplementary Table 3), no significant associations were seen in the NeuroX but nominally significant differences were identified for *AKT3* (P_skat_ = 0.049) and *BAP1* (P_skat_ = 0.004) in the exome cohort. A significant enrichment in *KIT* in the NeuroX was detected for rare, damaging nsSNPs (P_skat_ = 0.038) but was not seen in the exome data (P_skat_ = 0.571). A trend toward association was seen for *GRM* (P_skat_ = 0.075) in the Neurox data, and for *ERBB4* (P_skat_ = 0.055) and *TYR* (P_skat_ = 0.065) in the exome data. Although none survived correction for multiple testing, it does suggest possible case-control differences.

### 3.2 Case-control rare variant enrichment analysis

Dominant and recessive models were applied to LOF and/or nsSNPs variants within both PD data sets. Under a dominant model, no significant enrichment of carriers of rare nsSNPs was detected in the exome data. However, there was a nonsignificant increase in the number of carriers of dominant rare nsSNPs in 8 and 3 genes in both cases and controls, respectively. Similar results were seen in the NeuroX data, with 4/23 genes with suspected enrichment in cases and 4/23 genes in controls (Table 2). An increased number of carriers of rare *KIT* nsSNPs was detected in controls in NeuroX (OR_log_ = 0.68, 0.48–0.97, P_logreg_ = 0.035). The increased carrier frequency in controls is not consistent with the SKAT analysis, which suggested an increased burden of rare variants in cases. Two genes, *MITF* and *TP53*, also had increased number of carriers of rare alleles in controls compared to cases. Individual variant analysis within both data sets showed that no single variant within these genes was statistically enriched in controls (Supplementary Tables 4 and 5).

Two genes, *MAP2K2* and *PTEN*, had enrichment of carriers of dominant variants in cases in both cohorts, suggesting that these variants could increase PD risk; although this analysis again relates to very rare alleles. Despite this, on an individual scale, all observed dominant variants within these 2 genes in both data sets also appeared enriched in cases (Supplementary Tables 4 and 5).

Under a recessive model, although biallelic carriers of rare-damaging variants within the exome data were only seen in *DCC*, no significant difference was observed. In the NeuroX data, biallelic carriers appeared enriched in 4 genes, including *DCC* and *ERBB4*, although none reached significance (Table 3). No association with PD was detected for any individual rare variant in either cohort.

### 3.3 Rare variant meta-analyses

The overlap between the NeuroX and exome data is limited to 70 variants. Meta-analyses revealed a single significant association with the p.A421A/splice variant in *DCC* (OR_meta_ = 0.87, 0.76–0.99; P_meta_ = 0.047) but was not significant following correction for multiple testing correction (Supplementary Table 6).

Although no other significant association was seen, several variants were seen having ORs >2. The very rare p.V275F variant in *TYR* (encoding Tyrosinase) appears to have the largest effect (OR_meta_ = 4.13, 0.72–23.62). Although this carrier frequency of this variant was higher in cases than controls in both cohorts (NeuroX: 0.12% vs. 0.02%; exome: 0.08% vs. 0%), we had limited statistical power (further suggested by very large CIs) to find a significant association even on combining cohorts (9/8095; 0.11% vs. 1/6533; 0.02%), which is likely due to the rareness of the p.V275F variant. This very rare variant was corroborated by Sanger sequencing in 5/5 samples available (Supplementary Fig. 1). To further explore the role of p.V275F, we assessed carrier frequencies from an additional cohort of 642 PD exomes as well as data from ExAc consortium as a replication step (http://exac.broadinstitute.org/) (Supplementary Table 7). Consistent with the other cohorts, there was an excess of V275F carriers in cases compared to controls (0.16% vs. 0.02%). Including this data, meta-analysis demonstrated that >5-fold increased PD risk in p.V275F carriers (OR_meta_ = 5.42, 1.44–20.41; P_meta_ = 0.012) (Supplementary Fig. 2). p.V275F was also investigated in CMM exome sequencing data, and no difference between CMM cases and controls was observed (2/1298; MAF = 0.08% vs. 1/685; MAF = 0.07%). Although preliminary, the data suggests that the p.V275F variant may have a role in PD etiology.

### 3.4 Known melanoma variant analysis

Four and 5 variants definitively linked with CMM (germline or somatically) were present in exome and NeuroX data respectively. These variants were removed but had little effect on dominant and recessive models for all genes (data not shown). Individually, none of these variants were significantly associated with PD. Meta-analyses of the 2 variants present in both cohorts (p.A1276G in *GRIN2A* and p.E318K in *MITF*) were not found to influence PD risk.

## 4 Discussion

Epidemiological evidence has consistently suggested a shared susceptibility to CMM and PD (Bajaj et al., 2010, Becker et al., 2010, Catalá-López et al., 2014, D'Amelio et al., 2004, Elbaz et al., 2002, Elbaz et al., 2005, Gao et al., 2009a, Gao et al., 2009b, Kareus et al., 2012, Olsen et al., 2005, Olsen et al., 2006, Ong et al., 2014, Wirdefeldt et al., 2014). In this study, we investigated the role of rare variants in 29 CMM genes in PD risk using 2 large independent cohorts (exome-sequenced and SNP-genotyped) of PD cases and controls. Although our study is underpowered, as indicated by large confidence intervals, we have not identified a definitive overlap in genetic susceptibility in this large sample set. There was no increased burden of rare variants across all genes in either cohort. Gene-based comparisons identified a significant enrichment of rare, damaging *KIT* variants in cases in the NeuroX, data but this did not withstand correction for multiple testing. There was a trend toward enrichment of rare damaging variants in *GRM* in the NeuroX cases, and *ERBB4* and *TYR* in exome cases. There was no enrichment of rare nsSNPs in either cohort under a dominant model; however, there was a trend toward increased risk for *MAP2K2* and *PTEN*; and a trend toward reduced risk for *MITF* and *TP53*. Recessive biallelic carriers of *DCC* variants appeared over-represented in both cohorts. Interestingly, biallelic carriers of *ERBB4* variants were seen in 4/6875 NeuroX cases and 0/6605 controls (Supplementary Table 8). Dominant mutations in *ERBB4* have previously been shown to cause amyotrophic lateral sclerosis (OMIM #615515) (Takahashi et al., 2013) and therefore represents an attractive candidate gene for neurodegenerative disease in which recessive rare mutations may be linked to PD. Numerous individual variants were more common in cases rather than controls in either/both cohorts, with several genes showing high odds ratios on meta-analysis, although none was statistically significant.

Many rare moderate to high penetrant gene mutations cause PD (e.g., *LRRK2* and *PARK2*) or substantially increase PD risk (e.g., *GBA*) (Lubbe and Morris, 2014, Sidransky and Lopez, 2012). We investigated whether our results are modified by the presence/absence of known PD-linked variants within *LRRK2*, *PARK2*, and *GBA*. Removal of *LRRK2*, *PARK2*, or *GBA* variant carriers from both our dominant and recessive models did not change the overall results. No known PD mutations were found in any of the *TYR* p.V275F carriers.

Poor statistical power is an issue for all studies designed to uncover the genetics of complex diseases. It is becoming more evident that this is an issue for studies aimed at rare variant identification, and it has been proposed that studies such as this might need in excess of 25,000 discovery cases before adequate power is achieved (Zuk et al., 2014). Based on MAF = 1%, the exome data set only had 18.7% power to detect a 2-fold enrichment, and even lower power for even rarer MAFs. The NeuroX platform has been successfully used to identify several causative mutations in PD and other neurodegenerative diseases (Ghani et al., 2015). Despite being well powered to detect associations, the failure to replicate findings using the NeuroX platform is likely due to the fact that variants are preselected to focus specifically on genes/variants of interest to neurological disease (Nalls et al., 2015) and may therefore not be an accurate representation of variation across the CMM genes studied.

The very rare TYR p.V275F variant was more common in PD than controls in 3 independent data sets but not in CMM. Although all *TYR* p.V275F samples were successfully validated by Sanger sequencing, another potential concern is that not all variants investigated were confirmed by Sanger sequencing. This is particularly relevant to those genes which have a recognized pseudogene (e.g., *BRAF* and *PTEN*); however, stringent removal of reads mapping to other exonic locations would have restricted the inclusion of less secure genotypes in our analyses. The use of different capture methods in the exome data set represents a potential source of bias; however, stringent QC limited reads and variants seen in both captures across all samples thereby ensuring comparable high-quality genotype data. In addition, any systematic genotype bias should be present throughout the entire cohort as the same capture method was used for both cases and controls. The ExAc data represent samples collected for disease- and population-specific genetic studies and may therefore contain individuals with undiagnosed PD, which is likely to reduce any case-control differences. This suggests that our observed estimate may represent the lower bound of the true association.

The identification of a number of PD cases with the rare *TYR* p.V275F variant is of interest. Pigmentation genes contribute to CMM risk and have recently been proposed to contribute to PD (Double et al., 2010, Gao et al., 2009b, Herrero Hernández, 2009a, Herrero Hernández, 2009b, Pan et al., 2011). The exact role of *TYR* and other pigmentation genes in CMM has yet to be fully elucidated. TYR activity correlates well with skin color with biallelic variants, including p.V275F, causing recessive oculocutaneous albinism 1B (OCA1B, OMIM #606952) due to reduced TYR activity. None of the carriers were found to harbor additional OCA1B-associated mutations and are unlikely to have albinism. Known to be expressed in the human substantia nigra (Xu et al., 1997), tyrosinase is the rate-limiting enzyme in neuromelanin production as well as being responsible for the oxidation of L-tyrosine into L-DOPA during dopamine synthesis (Fedorow et al., 2005, Pan et al., 2011). It has been proposed that neuromelanin has neuroprotective effects by preventing the accumulation of toxins (Zecca et al., 2003). So, reduced TYR activity may contribute to the loss of neurons due to increased cell toxicity independent of α-synuclein (Hasegawa et al., 2006). Dopamine receptor activation has been shown to reduce dopaminergic neuron death (Nair et al., 2003, Vaarmann et al., 2013). Therefore, reduced dopamine production may predispose nigral neurons to apoptosis. The observed enrichment of p.V275F in PD cases along with the recent demonstration that rare variants in *GCH1* are associated with PD (Mencacci et al., 2014) provide further support for variation in the dopamine-biosynthetic pathway as being relevant to neurodegenerative PD.

## 5 Conclusions

Evidence for a shared genetic background between CMM and PD has been provided by epidemiological studies. Biological evidence of an overlap between the 2 diseases is further suggested by the fact that melanocytes and neurons of the substantia nigra are both pigmented cells derived from the neural crest (Gilbert, 2000); as well as that mitochondrial dysfunction is already implicated in both diseases (Devine et al., 2011). Based on our study, the role of rare variants in CMM genes in PD etiology appears limited. However, the observed excess of carriers of the very rare *TYR* variant p.V275F in PD cases in 3 independent cohorts suggests an involvement in disease pathogenesis and strengthens previous proposals linking pigmentation genes to PD. In addition, the prospect of unidentified changes, genetic or epigenetic, in unknown genes conferring increased risk for both diseases cannot be excluded and remains to be further investigated.

## Disclosure statement

Donald G. Grossett received personal fees from AbbVie, Union Chimique Belge Pharma, GE Healthcare, Civitas Inc, and Acorda Inc, outside the submitted work. Huw R. Morris reports personal fees from Teva, personal fees from Abbvie, personal fees from Teva, personal fees from UCB, personal fees from Boerhinger-Ingelheim, personal fees from GlaxoSmithKline, outside the submitted work. The remaining authors have no actual or potential conflicts of interest to declare.

## Figures and Tables

**Table 1:** Gene set–based burden analysis for the types of rare variants observed across all studied cutaneous malignant melanoma genes in the Parkinson's NeuroX and exome cohorts

Gene set tested	NeuroX		Exome	
	N	P_skat_	N	P_skat_
All variation (incl. synonymous)	237	0.756	554	0.403
All variation (excl. synonymous)	224	0.724	286	0.790
All rare^a^	206	0.948	269	0.693
Germline^b^	65	0.913	57	0.599
Somatic	197	0.665	255	0.795
All LOF variants	17	0.156	17	0.951
Rare LOF variants	16	0.321	16	0.999
All nsSNPs	215	0.839	269	0.682
Rare nsSNPs	198	0.963	253	0.586
All damaging nsSNPs	67	0.794	148	0.448
Rare-damaging nsSNPs	65	0.657	145	0.375
All damaging nsSNPs and LOFs	76	0.467	165	0.316
Rare damaging nsSNPs and LOFs	67	0.444	162	0.656

Gene set–based SKAT analysis assessing the burden of rare variants in all candidate cutaneous malignant melanoma genes in Parkinson's disease cases and controls in 2 large independent cohorts from the International Parkinson's Disease Genomics Consortium.

Key: incl., including; excl., excluding; LOF, loss of function; N, number of variants assessed; nsSNPs, nonsynonymous single nucleotide polymorphisms; P_skat_, *p*-values generated from SKAT analyses, correcting for gender, coverage metrics and principal components (1–4).

a Variants were classified as rare if their minor allele frequency was below 1%.

b If germline variants have been described to be associated with melanoma then it is labeled “Germline”.

**Table 2:** Comparison of the number of cases and controls, who harbor dominant rare nonsynonymous variants in the studied cutaneous malignant melanoma genes in the NeuroX and exome Parkinson's disease case-control cohorts

Gene	NeuroX							Exome						
	Cases (n = 6875)		Controls (n = 6065)		OR	95% CI	P_loreg_	Cases (n = 1255)		Controls (n = 475)		OR	95% CI	P_loreg_
	Carriers	Freq	Carriers	Freq				Carriers	Freq	Carriers	Freq			
*AKT3*								1	0.0007	1	0.0021	1.28	0.08–20.76	0.864
*BAP1*	*7*	*0.0010*	*10*	*0.0016*	*0.59*	*0.22–1.55*	*0.284*	4	0.0031	2	0.0042	0.95	0.15–6.14	0.953
*BRAF*								**3**	**0.0023**	**1**	**0.0021**	**1.64**	**0.16–16.60**	**0.675**
*CDK4*	**14**	**0.0020**	**8**	**0.0013**	**1.49**	**0.62–3.59**	**0.370**	0	0	4	0.0084			
*CDKN2A*	*37*	*0.0054*	*42*	*0.0069*	*0.75*	*0.48–1.17*	*0.210*	8	0.0063	3	0.0063	1.18	0.27–5.15	0.826
*DCC*	198	0.0288	193	0.0318	0.91	0.74–1.11	0.334	58	0.0462	27	0.0570	0.83	0.49–1.40	0.478
*EPHA2*	210	0.0305	199	0.0328	0.91	0.75–1.12	0.380	56	0.0446	21	0.0443	0.86	0.49–1.48	0.579
*ERBB4*	50	0.0073	41	0.0068	1.02	0.67–1.54	0.942	17	0.0135	9	0.0190	0.63	0.26–1.55	0.316
*GNA11*								1	0.0007	1	0.0021	0.18	0.01–3.97	0.278
*GNAQ*														
*GRIN2A*	254	0.0369	237	0.0391	0.94	0.78–1.12	0.490	74	0.0589	20	0.0422	1.08	0.63–1.87	0.774
*GRM3*	**12**	**0.0017**	**5**	**0.0008**	**2.13**	**0.74–6.10**	**0.161**	6	0.0047	3	0.0063	0.78	0.18–3.34	0.741
*HRAS*								**2**	**0.0015**	**0**	**0**			
*KIT*	*55*	*0.0080*	*70*	*0.0115*	*0.68*	*0.48–0.97*	*0.035*	*19*	*0.0151*	*10*	*0.0211*	*0.61*	*0.27–1.40*	*0.241*
*MAP2K1*								**2**	**0.0015**	**0**	**0**			
*MAP2K2*	**3**	**0.0004**	**1**	**0.0002**	**2.08**	**0.22–20.03**	**0.526**	**1**	**0.0007**	**0**	**0**			
*MC1R*	299	0.0435	254	0.0419	1.04	0.87–1.23	0.678	**4**	**0.0031**	**1**	**0.0021**	**2.02**	**0.22–18.50**	**0.536**
*MDM2*	15	0.0022	8	0.0013	1.52	0.64–3.61	0.339							
*MITF*	*37*	*0.0054*	*41*	*0.0068*	*0.77*	*0.49–1.21*	*0.258*	10	0.0079	5	0.0105	0.89	0.28–2.86	0.846
*NRAS*								**1**	**0.0007**	**0**	**0**			
*PDGFRB*	213	0.0310	178	0.0293	1.05	0.86–1.29	0.625	44	0.0350	17	0.0359	1.04	0.56–1.94	0.909
*PTEN*	**2**	**0.0003**	**1**	**0.0002**	**1.63**	**0.15–18.19**	**0.693**	**1**	**0.0007**	**0**	**0**			
*STK11*	52	0.0076	51	0.0084	0.88	0.59–1.30	0.518	**1**	**0.0007**	**0**	**0**			
*TERT*	70	0.0102	53	0.0087	1.14	0.79–1.63	0.487							
*TP53*	*4*	*0.0006*	*10*	*0.0016*	*0.36*	*0.11–1.16*	*0.087*	*5*	*0.0039*	*2*	*0.0042*	*0.57*	*0.09–3.68*	*0.552*
*TRRAP*	49	0.0071	49	0.0081	0.87	0.58–1.30	0.505	38	0.0302	12	0.0253	1.07	0.52–2.21	0.846
*TYR*	28	0.0041	19	0.0031	1.32	0.74–2.38	0.348	25	0.0199	11	0.0232	0.93	0.43–2.01	0.856
*XRCC3*	16	0.0023	10	0.0016	1.39	0.63–3.08	0.418	*2*	*0.0015*	*2*	*0.0042*	*0.54*	*0.07–4.20*	*0.553*
*ZNF831*	105	0.0153	96	0.0158	0.97	0.73–1.29	0.840							

The number of cases harboring rare damaging^a^ dominant-acting variants were compared against that observed in the controls in both the Neurox and exome data sets.

Values in italics represent genes which have more carriers in controls than in cases.

Values in bold represent genes which have more carriers in cases than in controls.

Key: carriers, number of carriers of dominant rare damaging variants; CI, confidence intervals; Freq, frequency; n, number of samples; OR, odds ratio; P_loreg_, *p*-values generated from logistic regression, correcting for gender, coverage metrics, and principal components (1–4).

^a^Damaging variants are defined as all loss of function variants and those predicted to be deleterious by ≥4 of the 6 in silico tools used.

**Table 3:** Cutaneous malignant melanoma genes demonstrating suggestive enrichment of cases harboring biallelic rare damaging^a^ variants under a recessive model in the NeuroX and exome Parkinson's disease case-control cohorts

Gene	Exome							NeuroX						
	Cases (n = 1255)		Controls (n = 475)		OR	95% CI	P_loreg_	Cases (n = 6875)		Controls (n = 6065)		OR	95% CI	P_loreg_
	Carriers	Freq	Carriers	Freq				Carriers	Freq	Carriers	Freq			
*DCC*	3	0.0024	1	0.0021	1.54	0.14–16.96	0.726	2	0.0003	1	0.0002	1.60	0.14–17.75	0.702
*EPHA2*	—	—	—	—	—	—	—	2	0.0003	1	0.0002	1.52	0.14–16.98	0.733
*ERBB4*	—	—	—	—	—	—	—	4	0.0006	0	0	—	—	—
*MITF*	—	—	—	—	—	—	—	1	0.0001	0	0	—	—	—

The number of cases harboring biallelic (homozygous or compound heterozygous) rare damaging^a^ variants were compared against that observed in the controls in both the Neurox and exome data sets.

Key: carriers, number of carriers of biallelic rare damaging variants; CI, confidence intervals; Freq, frequency; n, number of samples; OR, odds ratio; P_loreg_, *p*-values generated from logistic regression, correcting for gender, coverage metrics, and principal components (1–4).

a Damaging variants are defined as all loss of function variants and those predicted to be deleterious by ≥4 of the 6 in silico tools used.
