# Supplementary material for: Rare variants analysis of cutaneous malignant melanoma genes in Parkinson's disease
Source: Neurobiol Aging. 2016 Dec;48:222.e1–7. doi: 10.1016/j.neurobiolaging.2016.07.013 (PMC5096891; doi:10.1016/j.neurobiolaging.2016.07.013)
Supplement: Supplementary Figs. 1 and 2 [file mmc1.docx]

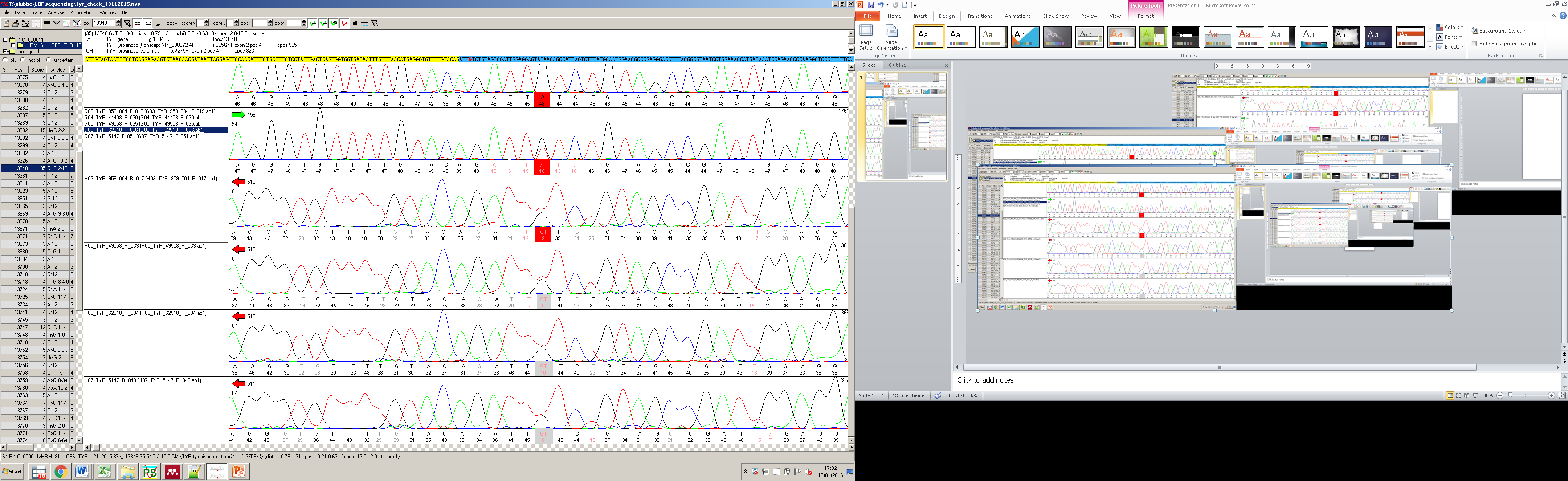

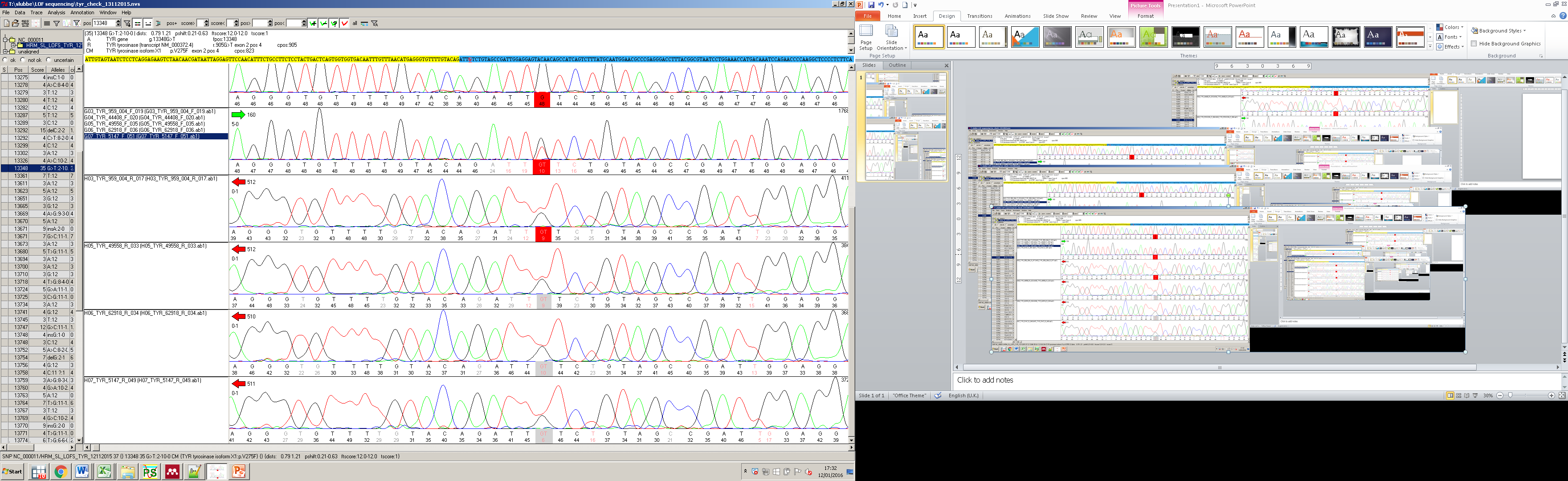

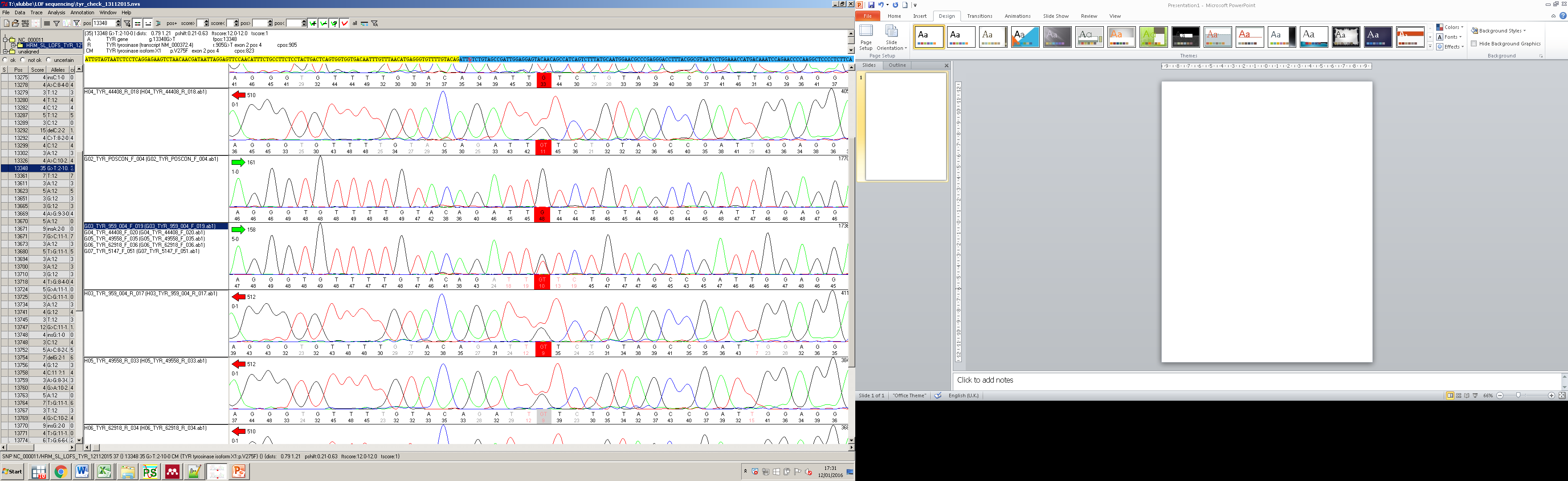

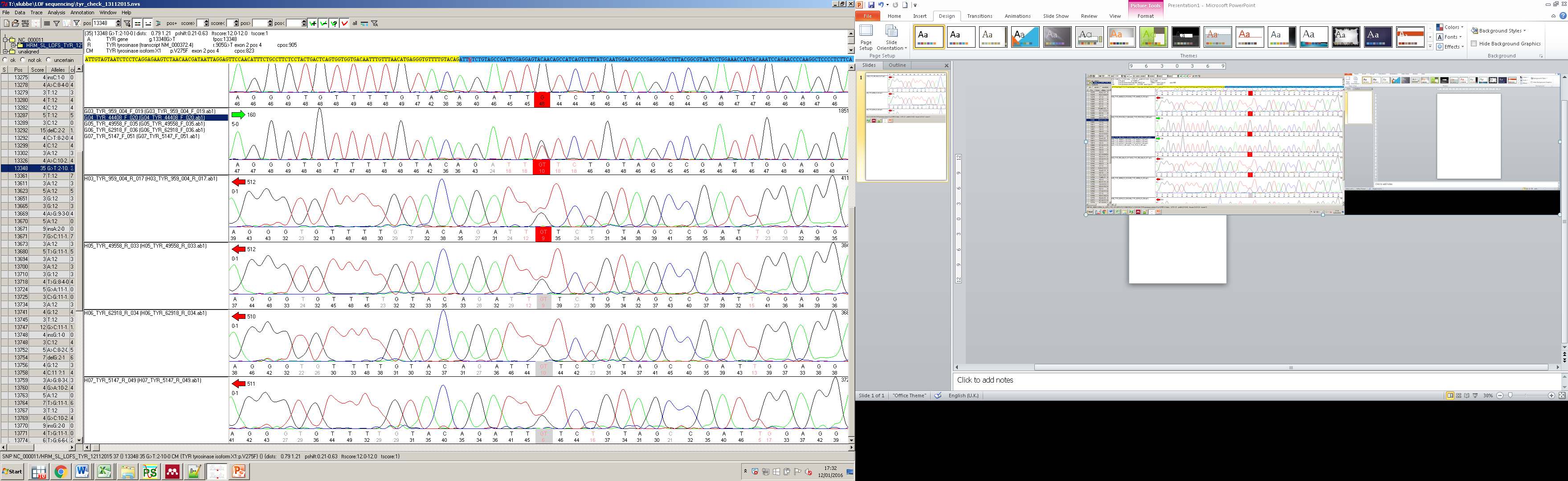

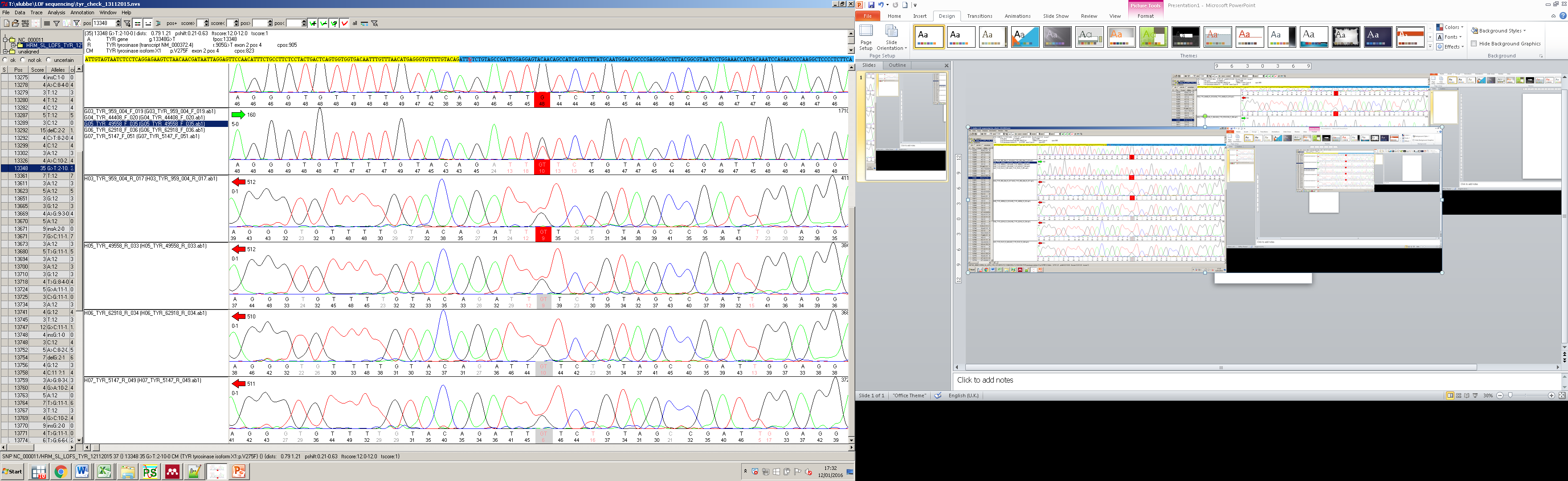


p.V275F Negative control

959_004

44408

49558

62918

5147

**Supplementary Figure 1:** Sanger sequence confirmation of the tyrosinase (*TYR*) p.V275F variant.

Overall (I-squared = 0.0%, p = 0.581)

Exomes-UCL

Cohort

NeuroX-IPDGC

Exome-IPDGC

5.42 (1.44, 20.41)

7.87 (1.02, 60.58)

OR (95% CI)

7.06 (0.88, 56.50)

1.15 (0.05, 28.40)

100.00

42.19

% Weight

40.67

17.14

1.0

0.1

0.5

2.0

4.0

Supplementary Figure 2: Forest plot of the odds ratio (OR) of Parkinson’s risk associated with the TYR p.V275F variant. Boxes denote OR point estimates, their areas proportional to the inverse variance weight of the estimate. Horizontal lines represent 95% CIs. Key: CI, confidence intervals; IPDGC, International Parkinson’s Disease Genomics Consortium; OR, odds ratio; UCL, University College London
